# Infection par le VIH chez les patientes atteintes de cancer du sein en Guinée (Afrique de l'Ouest)

**DOI:** 10.11604/pamj.2015.21.261.7146

**Published:** 2015-08-07

**Authors:** Bangaly Traore, Solomana Diane, Mamadou Saliou Sow, Mamady Keita, Mamoudou Conde, Fodé Amara Traore, Tidiane Kourouma

**Affiliations:** 1 Unité de Chirurgie Oncologique de Donka, CHU de Conakry, Guinée; 2 Service des Maladies Infectieuses et Tropicales de Donka, CHU de Conakry, Guinée

**Keywords:** Cancer du sein, VIH, diagnostic, traitement, breast cancer, HIV, diagnosis, treatment

## Abstract

L'objectif était de déterminer la prévalence de l'infection à VIH chez les patientes atteintes de cancer du sein et de comparer les caractéristiques anatomocliques et thérapeutiques de ces cancers du sein par rapports aux patientes non infectées par le VIH. Il s'agissait d'une étude rétrospective et analytique comparant les dossiers de patientes atteintes de cancers du sein histologiquement confirmés, infectées ou non par le VIH à l'unité de chirurgie oncologique de Donka, CHU de Conakry, de 2007 à 2012. Nous avons colligé 278 patientes présentant un cancer du sein dont 14 (5,0%) infectées par le VIH et 264 (95,0%) non infectées par le VIH. Les différences observées entre ces deux groupes de patientes étaient respectivement: âge médian (36,8 vs 49,0 ans), la ménopause (21,4% vs 53,4%), le nombre des patientes traitées (50,0% contre 77,1%) et la survenue de décès (78,6% vs 50,8%). Aucune différence n'a été notée dans la présentation clinique, histologique et le retard de consultation. Dans notre étude, la prévalence de l'infection à VIH chez les patients atteints de cancer du sein est élevée. L’âge jeune des patients, la faible accessibilité au traitement et la mortalité élevée doivent être confirmés par une étude sur un échantillon plus large.

## Introduction

L'infection par le virus de l'immunodéficience humaine (VIH) est une pandémie mondiale touchant particulièrement les pays en voie de développement. L'organisation mondiale de la santé (OMS) estime à 33.4 millions dont la majorité en Afrique subsaharienne [1]. En Guinée, le taux de prévalence était de 1,5% en 2012 [2]. De nombreux cancers ont vu leur incidence augmenter depuis l'avènement du VIH. On distingue les cancers classant SIDA (cancer du col utérin, sarcome de Kaposi, Lymphome malin non hodgkinien) des cancers non classant SIDA (cancer de l'anus, du foie, bronchopulmonaire …) [3]. Le lien entre le cancer du sein et le VIH n'est pas clairement établi. Il semble avoir une diminution du risque de cancer du sein chez les patientes infectées par le VIH. En effet, des études menées en Afrique [4] et aux USA [5] ont estimé que le risque de survenue de cancer du sein chez les personnes infectées par le VIH est faible par rapport à la population générale. Toutefois, l'association de ces deux maladies pose des problèmes de diagnostic et de prise en charge dans les pays à faibles ressources à cause du diagnostic tardif et de l'accès limité aux traitements. Le cancer du sein aurait-il un impact sur l'immunité des patientes infectées par le VIH ou les patientes infectées par le VIH ont-ils des cancers du sein différents de ceux chez des patientes non infectées par le VIH? C'est en répondant à ces questions dans notre contexte que cette étude a été réalisée avec pour objectifs de déterminer la prévalence de l'infection à VIH chez les patientes atteintes de cancer du sein et de comparer les caractéristiques anatomocliques et thérapeutiques de ces cancers du sein par rapports aux patientes non infectées par le VIH.

## Méthodes

Cette étude rétrospective et analytique a été menée à l'Unité de Chirurgie Oncologique de Donka, CHU de Conakry (Guinée) à partir du dossier des patientes atteintes de cancer du sein et infectées par le VIH de 2007 à 2012. Les dossiers de patientes souffrant de cancer du sein et non infectées par le VIH ont été retenus pour des fins de comparaison. La distribution par rapport à l’âge a été décrite. Le type histologique, le grade histopronostique, la classification TNM, le stade et le décompte du taux de CD4 au moment du diagnostic ont été les méthodes d’évaluation initiale. Avant le diagnostic de cancer du sein, certaines patientes étaient déjà sous traitement antirétroviral et d'autres l'ont été après. Une chimiothérapie néoadjuvante a été proposée à cause du diagnostic tardif du cancer. En cas de réponse objective, une mastectomie radicale avec curage axillaire était proposée, suivie d'un traitement adjuvant par chimiothérapie associée ou non à la radiothérapie. Les patientes ont été suivies et la médiane de survie a été calculée. Ces caractéristiques sociodémographiques, cliniques et thérapeutiques ont été comparées avec les patientes atteintes de cancer du sein non infectées par le VIH. Le test anova a été utilisé pour les variables quantitatives, les chi 2 de Pearson ou de tendance ont permis de tester les variables qualitatives. Le test était significatif si p est inférieur à 0,05.

## Résultats

De 2007 à 2012, nous avons colligé 278 dossiers de patientes atteintes de cancers du sein dont 14 (5,0%) infectées par le VIH et 264 (95,0%) non infectées par le VIH. L’âge médian était de 36,8 ans (36-40) contre 49 ans (20-85) pour les patientes non infectées par le VIH (p=0,002). Les personnes infectées par le VIH étaient ménopausées dans 21,4% versus 50,4% pour les non infectées (p=0,019). Nous n'avons pas noté de consommation de tabac ou d'alcool dans la population de femmes infectées par le VIH. Dans le groupe des patientes infectées par le VIH, le diagnostic de cancer était cytologique dans 8 cas (57,1%) et dans les 6 autres cas (42,9%) basé sur l'examen histologique de pièce opératoire ou de biopsie. Les types histologiques étaient: carcinome sans autre indication 10 cas, carcinome canalaire infiltrant 3 cas et lymphome de Burkitt 1 cas. Au moment du diagnostic, le taux de CD4 dosé était supérieur à 350 éléments/ml pour 4 patients sur 6. La tumeur était classée T4 dans 12 cas (85,7%) et métastatique dans 7 cas (50,0%). Le diagnostic de cancer du sein était tardif pour les patientes infectées par le VIH dans 92,9% contre 88,3% pour les patientes non infectées par le VIH (p=O,504). La chimiothérapie néoadjuvante a été réalisée dans 6 cas chez les patientes infectées par le VIH. Les protocoles utilisés étaient AC75 (doxorubucine + cyclophosphamide) dans 5 cas et AT (doxorubicine + docétaxel) dans 1 cas. La réponse clinique était complète pour une seule patiente qui présentait un cancer du sein classé T3N1M0 et partielle pour 2 patients dont le cancer était classé T4bN1M0. Dans 1 cas, une deuxième ligne par docétaxel a dû être utilisée pour avoir une réponse clinique partielle. Les complications infectieuses liées à la chimiothérapie étaient 2 cas d'infections cutanées et digestives sans neutropénie et 2 cas de neutropénie sévère associées à une candidose digestive. La mastectomie avec curage ganglionnaire axillaire a été réalisée pour ces patientes au nombre de trois (3). Les suites opératoires ont été simples jusqu’à cicatrisation. Deux patientes ont bénéficié de la chimiothérapie. La seule patiente qui a eu la réponse complète avait bénéficié d'une radiothérapie adjuvante. Les trois malades opérées ont présenté une récidive locorégionale et métastatique après des délais respectifs de 1, 2 et 21 mois, soit un délai médian de récidive de 2 mois. Le délai médian de suivi était de 5 mois (0-54) contre 11 mois (0-71 pour les patientes non infectées par le VIH (p=0,1). A la date de point, 11 (78,6%) patientes infectées par le VIH sont décédées versus 134 (50,8%) de décès parmi les patientes non infectées par le VIH (p= 0,042). Le Tableau 1 présente les principales caractéristiques cliniques, thérapeutiques et évolutives des 14 patientes comparées aux 264 patientes non infectées par le VIH.

**Tableau 1:** Comparaison des caractéristiques sociodémographiques, diagnostique et thérapeutique des cancers du sein chez des patientes infectées ou non par le VIH

Caractéristiques	Patientes infectées par le VIH (n=14)	Patientes non infectées par le VIH (n=264)	Valeur p
Age médian (extrêmes)	36,5 (26 - 58)	49 (20 - 85)	0,002+
IMC	22,5 (17 - 33)	24 (15 - 41)	0,529
Ménopause n(%)	3 (27,3%)	133 (50,4%)	0,03+
Délai médian de consultation en mois (extrêmes)	5 (1 - 12)	9 (1 - 120)	0,124
Atteinte cutanée n(%)	10 (71,4%)	152 (57,6%)	0,305
Inflammatoire n(%)	3 (21,4%)	50 (18,9%)	0,519
Stade précoce (I - II) n(%)	1 (7,1%)	31 (11,7%)	0,504
Stade tardif (III - IV) n(%)	13 (92,9%)	233 (88,3%)	
Métastases	7 (50,0%)	78 (25,5%)	0,09
Morphologie n(%)			
Carcinome canalaire infiltrant	3 (21,4%)	151 (57,2%)	0,138
Carcinome lobulaire infiltrant	-	13 (9,9%)	
Carcinome SAI	10 (74,2%)	75 (28,4%)	
Lymphome malin non hodgkinien	1 (7,1%)	1 (0,4%)	
Autres	-	30 (11,4%)	
Traitement n(%)	7 (50,0%)	202 (76,5%)	0,033+
Réponse à la chimiothérapie néoadjuvante n(%)			
Objective	2 (28,6%)	70 (65,4%)	0,06
Non objective	5 (71,4%)	37 (34,6%)	
Récidive n(%)	3 (100%)	48 (39,3%)	0,06
Suivi			
Médian en mois (extrêmes)	5 mois (0 - 54)	11 mois (0 - 71)	0,100
Perdu de vue n(%)	2 (14,3%)	44 (16,7%)	0,58
Décès n(%)	11(78,6%)	134 (50,8%)	0,042+

+ Non significatif

IMC = Indice de masse corporelle, SAI: Sans autre indication

## Discussion

La prévalence de l'infection à VIH chez les patients atteints de cancer du sein était de 5% dans notre étude. Cette prévalence est hospitalière et ne peut être comparée à celle de population générale en Guinée qui était de 1,5% en 2012 [2]. Elle était de 6% chez les patientes atteintes de cancer en milieu hospitalier tertiaire au Nigeria [6]. Le risque de cancer du sein chez les personnes infectées par le VIH est sujet de controverse. Pour certains auteurs [4, 5], il y'a une diminution du risque alors que pour d'autres [7], il y'a une augmentation du nombre de cancers de cancers non classant SIDA, incluant ceux du poumon, du sein et de l'anus. Une étude de cohorte des patientes infectées par le VIH pourrait aider à élucider cette question dans notre contexte. Dans cette étude, les femmes infectées sont plus jeunes et sont non menauposées par rapport aux femmes non infectées. L’âge médian dans l’étude de Spano JP et al. [8] en France était de 43,8 ans. A part l’âge et la ménopause, nous n'avons pas trouvé de différence statistiquement significative par rapport à l'indice de masse corporelle, la présentation clinique, le type histologique et le stade du cancer du sein. Les mêmes constats sont faits par Sarhan M [9] qui, en plus, n'a pas trouvé de différence entre l’âge de survenue de cancer dans les deux groupes; la survenue de cancer du sein avant la ménopause n’étant pas non plus associée à l'agressivité de la tumeur. Sur le plan histologique, les carcinomes (canalaires surtout) sont les plus fréquents [8], mais les cas de lymphomes non hodgkiniens sont aussi rapportés [10]. Dans notre série, il s'agissait à majorité de carcinomes et un cas de lymphome de Burkitt. Le taux de CD4 ne semble pas avoir d'influence sur la survenue ou le stade du cancer du sein [11] car le taux de CD4 était supérieur à 350 élts/ml dans 4 fois sur 6 au moment du diagnostic du cancer du sein. Les complications liées à la chimiothérapie sont essentiellement la neutropénie et les infections chez les patientes infectées par le VIH. Aucune complication post opératoire n'a été notée chez nos patientes en cours d'hospitalisation. La survie est également sujette de controverses. Selon Sarhan M [9], il n'y a pas de différence de survie pour cancer du sein entre personnes infectées par le VIH ou non. Pour d'autres [3, 8], les taux de survie sont significativement plus faibles que dans la population générale à cause de la sévérité de la présentation et de la complexité de la prise en charge. En plus, la mortalité élevée dans notre étude pourrait s'expliquer par la faible accessibilité aux traitements (antirétroviral et anticancéreux) des patients infectés par le VIH. La médiane de survie de 5 mois dans la population de personnes infectée par le VIH est inférieure à celle d'une cohorte brésilienne qui était de 12 mois [12]. Dans une étude faite en Ouganda, l'infection par le VIH augmente le risque de décès chez les patients souffrant de cancer [13].

## Conclusion

Dans notre étude, la prévalence de l'infection à VIH chez les patients atteints de cancer du sein est élevée. L’âge jeune des patients, la faible accessibilité au traitement et la mortalité élevée doivent être confirmés par une étude sur un échantillon plus large.

## References

[1] WHO (2009). WHO AIDS Epidemic Report 09.

[2] (2012). Rapport UNGASD Guinée. http://www.unaids.org/en/…/2012countries/ce_GN_Narrative_Report[1].pdf?.

[3] Lanoy E, Guiguet M (2010). Cancer et infection à VIH. Médecine sciences: M/S..

[4] Sasco AJ, Jaquet A, Boidin E (2010). The challenge of AIDS-related malignancies in Sub-saharan Africa. PloS ONE..

[5] Goedert JJ, Schairer C, McNee TS (2006). For the HIV/AIDS Cancer Match Study5 Risk of breast, ovary, and uterine corpus cancers among 85 268 women with AIDS. British Journal of Cancer..

[6] Ocheni S, Aken'Ova YA (2004). Association between HIV/AIDS and malignancies in a Nigerian tertiary institution. West Afr J Med..

[7] Shiels MS, Pfeiffer RM, Gail MH (2011). Cancer burden in the HIV-infected population in the United States. J Natl Cancer Inst..

[8] Spano JP, Lanoy E, Mounier N (2012). Breast cancer among HIV infected individuals from the ONCOVIH study, in France: Therapeutic implications. Eur J Cancer..

[9] Sarhan M, DePaz HA, Oluwole SF (2010). Breast cancer in women with human immunodeficiency virus infection: pathological, clinical, and prognosis implications. J Womens Health (Larchmt).

[10] Chanan-Khan A, Holkova B, Goldenberg AS, Pavlick A, Demopoulos R, Takeshita K (2005). Non-hodkin's lymphoma presenting as a breast mass in patients with HIV infections: a report of three cases. Leuk Lymphoma..

[11] Ruiz M, Davis H (2011). Breast cancer in HIV-Infected patients: A retrospective Simple-Institution Study. J Int Assoc Physians AIDS care (Chic).

[12] Andrade AC, Luz PM, Veloso VG (2011). Breast cancer in a cohort of human immunodeficiency virus (HIV)-infected women from Rio de Janeiro, Brazil: a cases series report and an incidence rate estimate. Braz J Infect Dis..

[13] Coghill AE, Newcomb PA, Madeleine MM (2013). Contribution of HIV infection to mortality among cancer patients in Uganda. AIDS.

